# Electronic and magnetism properties of two-dimensional stacked nickel hydroxides and nitrides

**DOI:** 10.1038/srep11656

**Published:** 2015-06-26

**Authors:** Xiao-Lin Wei, Zhen-Kun Tang, Gen-Cai Guo, Shangyi Ma, Li-Min Liu

**Affiliations:** 1 Hunan Key Laboratory for Micro-Nano Energy Materials and Device, Department of Physics, Xiangtan University, Hunan, 411105, China; 2 Beijing Computational Science Research Center, Beijing 10084, China; 3 Departments of Physics and Electronics, Hengyang Normal University, Hengyang 421008, China; 4 Chengdu Green Energy and Green Manufacturing Technology R&D Center, Chengdu, Sichuan, 610207, China

## Abstract

Two-dimensional (2D) layered materials receive a lot of attention because of their outstanding intrinsic properties and wide applications. In this work, the structural, electronic and magnetic properties of nickel hydroxides (Ni(OH)_2_) and nitrides XN (X = B, Al, and Ga) heterostructures are studied by first-principles calculations. The results show that the pristine monolayer Ni(OH)_2_ owns no macro magnetism with antiferromagnetic (AFM) coupling between two nearest Ni atoms, the electronic structure can be modulated through the heterostructures. The Ni(OH)_2_-GaN and Ni(OH)_2_-AlN heterostructures retain the AFM coupling, while Ni(OH)_2_-BN heterostructure have a larger magnetic moment with ferromagnetic (FM) coupling. The complete electron–hole separation is found in the Ni(OH)_2_-GaN heterostructure. The tunable electronic and magnetic properties of the Ni(OH)_2_-XN heterostructures open a new door to design the spintronic devices in the 2D stacked nanostructures.

Van der Waals (vdW) heterostructures^1^,^2^,^3^,^4^,^5^ especially for the layered heterostructures fabricated by stacking different 2D semiconductors have been the focus of research interest as promising materials for the design of new devices in photonics, electronics, and optoelectronics. To fabricate a 2D semiconducting hetero-system with the desired heterojunction type for the applications, many combinations of semiconductors have been widely examined^6^,^7^,^8^,^9^,^10^,^11^,^12^,^13^,^14^. Experimentally, various heterostructures have been developed by the junction of two other semiconductors like transition metal dichalcogenides^6^,^7^. Besides, the flexible electronic properties^8^,^9^,^10^,^11^,^12^ and fascinating optical properties^13^,^14^ of many novel 2D stacked layered heterostructures have been investigated by theoretical calculation. Recently, spin-based devices^15^,^16^,^17^,^18^,^19^,^20^,^21^,^22^ plays an extremely important role in the relatively novel field of microelectronic and computer sciences. The most exciting event in recent years may be the discovery of the giant magnetoresistance (GMR) effect in metallic multilayer films^23^,^24^ and the successful application of this effect to information storage. Furthermore, much of quasi-2D magnetic multilayers structures^25^,^26^,^27^,^28^ with unique physical properties are also achieved in the recent experiments. However, there is few theoretical work focus on the 2D magnetic heterostructures with stacking monolayer semiconductors.

Hexagonal Ni(OH)_2_ ultrathin nanosheets have synthesized by exfoliation of layered nickel hydroxides^29^ or simple electrochemical reaction^30^. Unfortunately, the magnetic ground state of monolayer Ni(OH)_2_ shows that this material has an antiferromagnetic (AFM) order without macro magnetic moment^31^,^32^. However, 2D heterojunctions interfacing different layered materials would enable the so-called van der Waals epitaxy^33^, in which the lattice matching condition in traditional epitaxy is drastically relaxed, allowing the formation of a novel layered heterostructures with some fascinating physical properties. Thus, it is great necessary to know whether the heterostructure can help to modulate electronic and magnetic properties.

In this work, first-principles calculations were carried out to systematically examine the structural, electronic and magnetic properties of the Ni(OH)_2_–XN heterostructures (X = B, Al, and Ga) with van der Waals (vdW) correction. The calculated results show that the ground state of the pristine monolayer Ni(OH)_2_ is a semiconductor with AFM coupling between two nearest Ni atoms. Interestingly, the magnetic coupling of Ni(OH)_2_ can be easily tuned by forming the heterostructure with monolayer XN. It is found that Ni(OH)_2_-GaN and Ni(OH)_2_-AlN heterostructures retain the AFM coupling between Ni atoms, while Ni(OH)_2_-BN heterostructure have a larger magnetic moment with ferromagnetic (FM) coupling between Ni atoms. More interestingly, complete electron–hole separation is found in the Ni(OH)_2_-GaN heterostructure. The proposed Ni(OH)_2_–XN heterostructures possess many novel properties, such as tunable magnetic coupling and electronic-hole separation, which enable them to have great potential applications for spintronics and photocatalysis.

## Results

**Figure 1:**
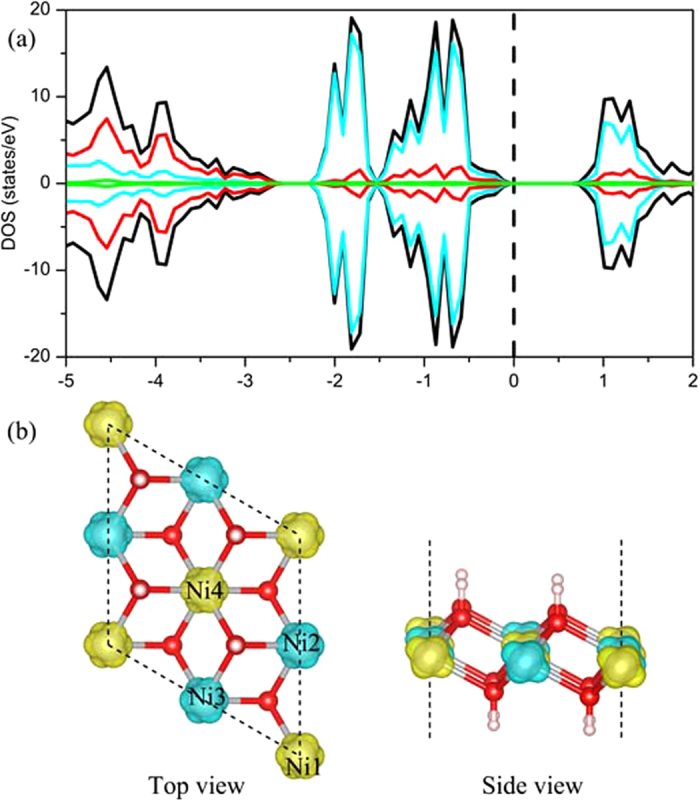
(**a**) The total and partial DOSs of the pristine monolayer Ni(OH)_2_. The black, glaucous, red, and green lines in the upper DOS images represent the total DOS, the partial DOS of Ni, O, and F atoms, respectively. (**b**) The spin density distributions of the of the pristine monolayer Ni(OH)_2_. The grey, red, pink, green, dark green, brown, and blue balls represent Ni, O, H, B, Al, Ga, and N atoms, respectively. The yellow and glaucous isosurfaces correspond to the spin up and spin down density, respectively (the isovalue is 0.05 a.u). In order to study the different magnetic coupling of Ni atoms, the different atoms of the unit cell are labeled as Ni1, Ni2, Ni3, and Ni4, respectively.

Our calculation results show that the monolayer Ni(OH)_2_ remain exhibit AFM coupling between two nearest Ni atoms, as shown in Fig. 1. The total energy of monolayer Ni(OH)_2_ with AFM coupling is 0.045 eV lower than that with FM coupling. In this work, three possible magnetic couplings: FM (Ni1↑, Ni2↑, Ni3↑, Ni4↑), AFM1 (Ni1↑, Ni2↓, Ni3↑, Ni4↓), and AFM2 (Ni1↑, Ni2↓, Ni3↓, Ni4↑) are considered. The different Ni atoms are labeled as Ni1, Ni2, Ni3, and Ni4, respectively (as shown in Fig. 1). The electronic density of states (DOS) in the Fig. 1 (a) show that the monolayer Ni(OH)_2_ is semiconductor without macro magnetic moment. Furthermore, it is worth noting that each Ni atom have 1.572 μ_B_ magnetic moment with AFM coupling, as shown in the Fig. 1 (b).

For a 2D material, its quasi-2D stacked structure, such as vdW heterostructure, always plays an important role to deliver its potential into practical applications. As mentioned in the introduction, in order to know whether the heterorstructure between the Ni(OH)_2_ and other 2D materials can tune the electronic properties of the Ni(OH)_2_, the typical heterostructure between the Ni(OH)_2_ and nitrides are explored. In the following, the electronic and magnetic properties of three types of stacked vdW heterostructures are investigated. As shown in our previous work^14^, the monolayer nitrides XN (X = B, Al, and Ga) are thermodynamically stable, and all of them are typical semiconductors with band gaps of 2.00–4.69 eV.

**Figure 2:**
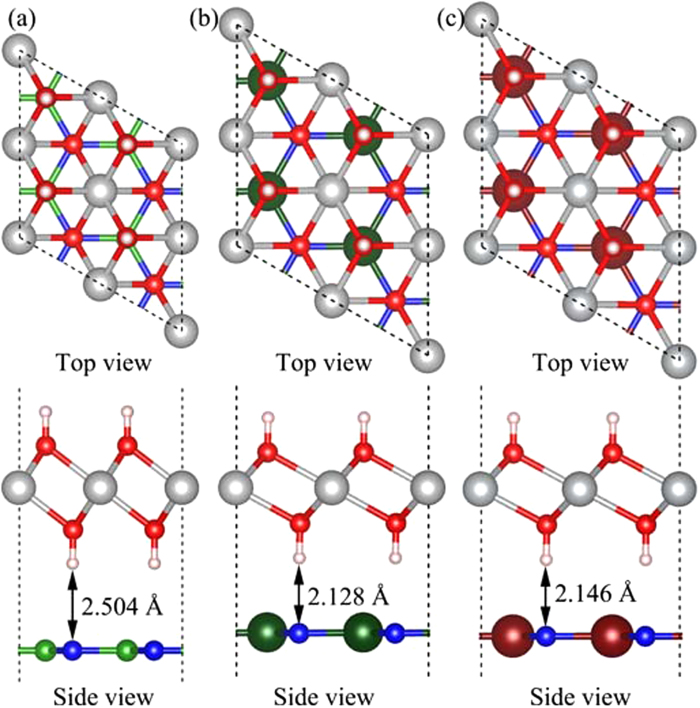
The relaxed atomic structures of the stacked (**a**) Ni(OH)_2_-BN, (**b**) Ni(OH)_2_-AlN and (**c**) Ni(OH)_2_-GaN heterostructures. The grey, red, pink, green, dark green, brown, and blue balls represent Ni, O, H, B, Al, Ga, and N atoms, respectively.

The bilayer heterostructures are constructed by stacking (2 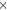 2 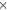 1) supercell Ni(OH)_2_ and monolayer XN (X = B, Al, and Ga), denoted as Ni(OH)_2_–XN for simplicity, as shown in Fig. 2. The calculated lattice constant of the monolayer Ni(OH)_2_ unit cells is 3.200 Å. The optimized lattice constants of the unit cells for Ni(OH)_2_–BN, Ni(OH)_2_–AlN and Ni(OH)_2_–GaN are 2.705 Å, 3.123 Å and 3.202 Å, respectively. Thus, the lattice mismatch between Ni(OH)_2_ and XN are 15.5%, 2.4% and 0.0% for X = B, Al and Ga, respectively. There is a larger lattice mismatch between Ni(OH)_2_ and BN, and this lattice mismatch may be achieved in nanoscale heterostructures^34^,^35^. Besides, the ground state geometries of Ni(OH)_2_–XN heterostructures are determined by the weak vdW interactions between neighboring layers. The equilibrium interlayer distance, d_Ni(OH)2/XN_, is defined as the distance between the H atoms in Ni(OH)_2_ layers and the neighboring XN planes. The calculated d_Ni(OH)2/XN_ for Ni(OH)_2_–XN are 2.504 Å, 2.128 Å, and 2.146 Å for X = B, Al and Ga, respectively.

**Figure 3:**
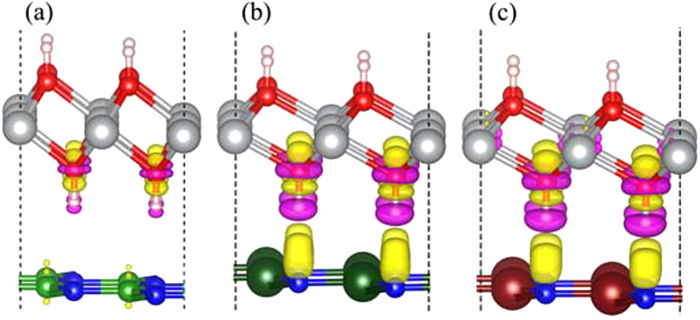
The corresponding charge density difference of the stacked (**a**) Ni(OH)_2_-BN, (**b**) Ni(OH)_2_-AlN and (**c**) Ni(OH)_2_-GaN heterostructures. The grey, red, pink, green, dark green, brown, and blue balls represent Ni, O, H, B, Al, Ga, and N atoms, respectively. The yellow and violet isosurfaces correspond to the accumulation and depletion of electronic densities (the isovalue is 0.001 a.u).

To understand the charge transfer of the heterostructures, the three-dimensional charge density differences are calculated by subtracting the calculated electronic charge of Ni(OH)_2_–XN from that of the independent monolayer Ni(OH)_2_ and XN. As shown in Fig. 3, the charge transfer at the interface of Ni(OH)_2_–BN is negligible, which agrees with the relatively large interfacial distance between Ni(OH)_2_ and BN. While, the electrons transfer from H atoms to N atoms at the interface with a large charge redistributions in the Ni(OH)_2_–AlN and Ni(OH)_2_–GaN heterostuctures. Such result suggests the Ni(OH)_2_–AlN and Ni(OH)_2_–GaN form the relatively strong adhesive interface.

The Ni atom is spin polarized in the monolayer Ni(OH)_2_ with AFM coupling. One may wonder whether the Ni(OH)_2_–XN heterostuctures have different magnetic coupling or tunable magnetic properties. Thus, we calculate the relative energy of different magnetic coupling, ground state geometries, and magnetic moment of Ni(OH)_2_–XN heterostructures as shown in Table 1. The calculated results show that the most stable state of the Ni(OH)_2_–BN heterostucture is FM state, which is about 61 and 59 meV per cell lower than the AFM1 and AFM2 states, respectively. The total magnetic moments are 7.914 μ_B_. While the total magnetic moments of the Ni(OH)_2_–AlN and Ni(OH)_2_–GaN heterostuctures are zero with AFM1 and AFM2 coupling, respectively. The larger lattice mismatch between Ni(OH)_2_ and BN induce a larger compressing stress at the interface, which result the change of the magnetic coupling between the Ni atoms. Considering the relatively large lattice mismatch of 15%, it may be not easy to experimentally fabricate the Ni(OH)_2_-BN. In order to know whether the AFM-FM transition can occur for the heterostructure with a small lattice constant mismatch. Another system, BeO is considered to form heterostructure with Ni(OH)_2_. The lattice mismatch between this two materials is about 10.3%, which is obviously smaller than the Ni(OH)_2_-BN. The further calculations show that the Ni(OH)_2_-BeO heterostructure also has the FM ground state, which is about 34 and 33 meV per cell lower than the AFM1 and AFM2 states, respectively. Compared with the energy differences (61/59 meV) between AFM1/AFM2 and FM coupling of Ni(OH)_2_-BN heterostructure with 15.5% lattice mismatch, we could speculate that the heterostructure with even smaller lattice mismatch (<10%) can lead an AFM-FM transition.

**Figure 4:**
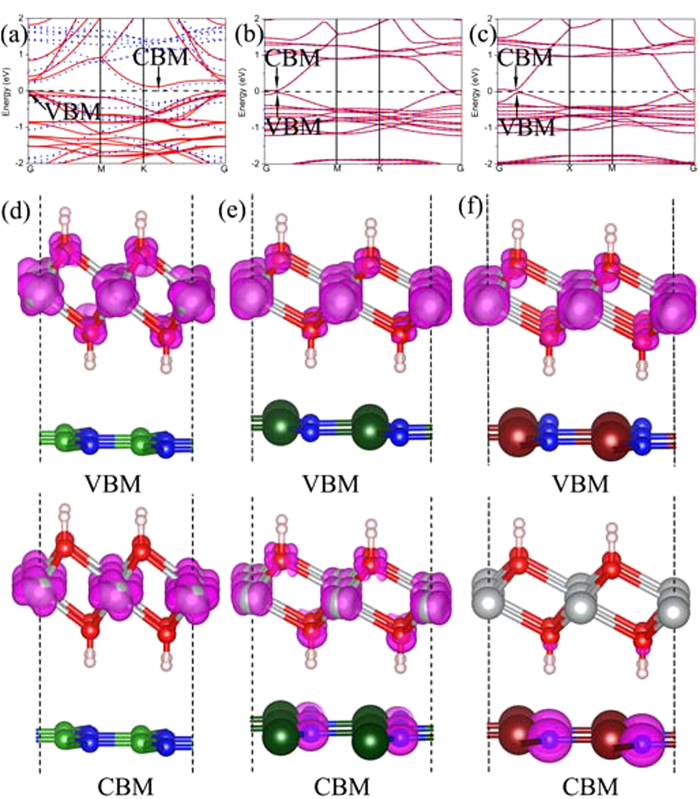
The band structures of the stacked (**a**) Ni(OH)_2_-BN, (**b**) Ni(OH)_2_-AlN and (**c**) Ni(OH)_2_-GaN heterostructures. The red solid lines and blue dashed lines in the band structures images represent the spin up bands and spin down bands, respectively. (**d–f**) The corresponding charge distribution of the valence band maximum (VBM) and the conduction band minimum (CBM) are shown on the upper and low panel in violet color as well (the isovalue is 0.01 a.u). The grey, red, pink, green, dark green, brown, and blue balls represent Ni, O, H, B, Al, Ga and N atoms, respectively.

It is well-known that both of monolayer Ni(OH)_2_ and XN are semiconductor. Thus it is necessary to know whether the Ni(OH)_2_–XN heterostuctures owns the unique electronic structure. In order to know the detailed electronic structures of the Ni(OH)_2_–XN heterostuctures, the calculated band structures are shown in Fig. 4 (a)–(c). The band structure in the Fig. 4 (a) indicates that the Ni(OH)_2_–BN is a magnetic semiconductor with a very small band gap (0.001 eV). The corresponding electronic densities of the valence band maximum (VBM) and the conduction band minimum (CBM) are plotted in Fig. 4 (d). It is found that the electronic densities of the VBM and the CBM are mainly distributed on the Ni atoms in the Ni(OH)_2_ layer. However, the band structures of Ni(OH)_2_–AlN and Ni(OH)_2_–GaN are obviously different from that of the Ni(OH)_2_–BN. The band gap of Ni(OH)_2_–AlN and Ni(OH)_2_–GaN is 0.060 eV and 0.063 eV, respectively. The VBM of the Ni(OH)_2_–AlN localize on the Ni atoms, while the CBM of the Ni(OH)_2_–AlN localize on the Ni, O, and N atoms in the interface, as shown in Fig. 4 (e). Interestingly, the complete electronic densities separation of VBM and CBM on Ni(OH)_2_ and GaN, respectively, in Ni(OH)_2_–GaN, as depicted more clearly in Fig. 4 (f). The electrons and holes separated in the Ni(OH)_2_–GaN heterostructures can suppresses charge recombination, which has potential applying in the photoelectric device^36^,^37^. It should be noted that the pure DFT usually underestimate the band gap of semiconductor, thus the real band gap of the heterostructure should be larger than the results mentioned above.

**Figure 5:**
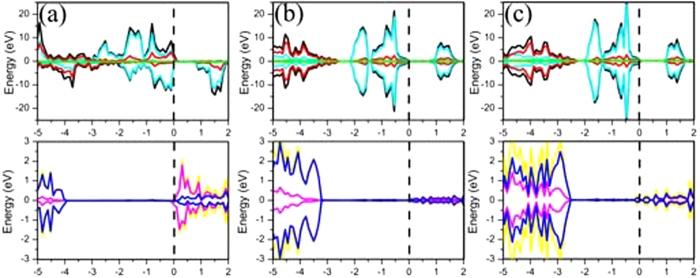
The DOS of the stacked (**a**) Ni(OH)_2_-BN, (**b**) Ni(OH)_2_-AlN and (**c**) Ni(OH)_2_-GaN heterostructures. The DOS of Ni(OH)_2_ and XN (X = B, Al, and Ga) are shown on the upper and low panel, respectively. The black, glaucous, red, and green lines in the upper DOS images represent the total DOS of Ni(OH)_2_, the partial DOS of Ni, O, and F atoms, respectively. The yellow, blue, and violet lines in the low DOS images represent the total DOS of XN, the partial DOS of N, and X (X = B, Al, and Ga) atoms, respectively.

To obtain deeper insight into the electronic structure of the Ni(OH)_2_–XN heterostuctures, the projected DOS of Ni(OH)_2_ and XN are analyzed in Fig. 5. The upper panel of Fig. 5 (a) show the total and projected DOS of Ni(OH)_2_ layer in Ni(OH)_2_–BN heterostuctures, which is a magnetic semiconductor with a small band gap. The spin-up DOS near the Fermi level is mainly provided by the Ni atom DOS and slightly provided by the O atom DOS. This means that the magnetic moment of the Ni(OH)_2_ layer is mainly located on the Ni atom. While, the low panel of Fig. 5(a) show the total and projected DOS of BN layer in Ni(OH)_2_–BN heterostuctures. The DOS curves indicate that BN layer is semiconductor with a lager band gap. It is obvious that the energy level of VBM is much lower than that of Ni(OH)_2_, which is the reason of charge transfer from the Ni(OH)_2_ layer to XN layer, as shown in the Fig. 3. In addition, there is no any overlap between Ni(OH)_2_ layer and XN layer due to the weak vdW interaction between them.

The calculated projected DOS in the Fig. 5 (b)–(c) clearly suggest that the Ni(OH)_2_–AlN and Ni(OH)_2_–GaN heterostuctures are semiconductor without obvious magnetic moment. The DOS of Ni(OH)_2_ layer in the Ni(OH)_2_–AlN and Ni(OH)_2_–GaN heterostuctures are quite close. There are little hybrid DOS in the band gap of AlN and GaN layer. More hybrid DOS in GaN layer means that GaN layer will be more affected by Ni(OH)_2_ layer than AlN layer. This is excellent agreement with the charge density difference distributions in Fig. 3.

**Figure 6:**
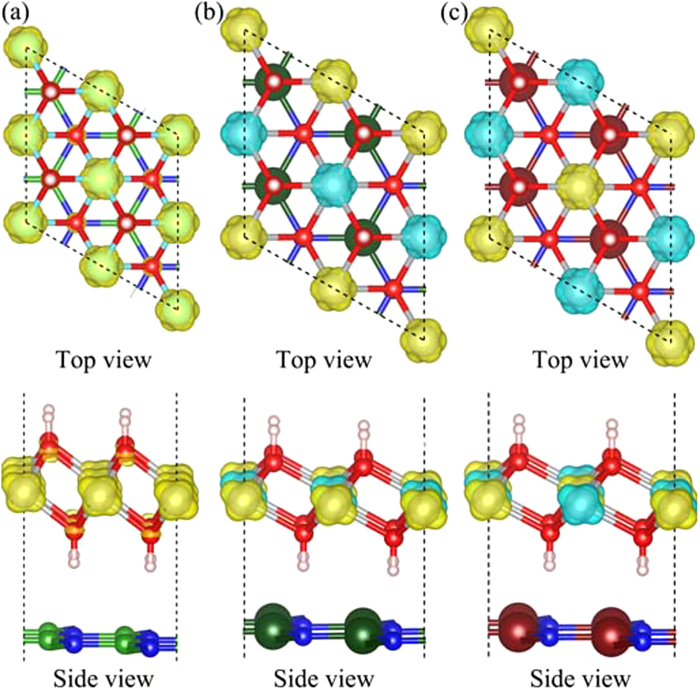
The spin density distributions of the of the stacked (**a**) Ni(OH)_2_-BN, (**b**) Ni(OH)_2_-AlN and (**c**) Ni(OH)_2_-GaN heterostructures. The grey, red, pink, green, dark green, brown, and blue balls represent Ni, O, H, B, Al, Ga, and N atoms, respectively. The yellow and glaucous isosurfaces correspond to the spin up and spin down density, respectively (the isovalue is 0.05 a.u).

Finally, the origin of magnetic moment of the Ni(OH)_2_–XN heterostuctures are examined by the distribution of the spin density, as shown in Fig. 6. The total magnetic moment of the Ni(OH)_2_–BN is 7.914 μ_B_ per spuercell in FM coupling. Each Ni atom at the Ni(OH)_2_–BN is 1.496 μ_B_, and the interfacial O atom at the Ni(OH)_2_–BN is 0.193 μ_B_. It is clear that the spin densities of the Ni(OH)_2_–XN heterostuctures are mainly localized on the Ni atoms with FM coupling, and slightly localized on the O atoms (see Fig. 6 (a)). Besides, the total magnetic moment of the Ni(OH)_2_–AlN is 0 μ_B_ per spuercell with AFM coupling. The Ni2 and Ni4 atom at the Ni(OH)_2_–AlN is 1.558 μ_B_, while the Ni1 and Ni3 atom at the Ni(OH)_2_–AlN is −1.558 μ_B_ (the different Ni atoms are labeled in Fig. 1 (b)), as shown in Fig. 6 (b). Fig. 6 (c) show that another AFM coupling in the Ni(OH)_2_–GaN with total magnetic moment is 0 μ_B_, The corresponding magnetic moment of Ni1 and Ni4 atom is 1.563 μ_B_, and the magnetic moment of Ni2 and Ni3 atom is −1.563 μ_B_. It is obvious that the magnitude of the magnetic moment of single Ni atom significantly changes with different lattice mismatch. More importantly, when a larger lattice mismatch existsin the Ni(OH)_2_–BN heterostucture, a typical FM coupling occurs with 7.914 μ_B_ magnetic moment per supercell. The tunable magnetism of the Ni(OH)_2_–XN heterostuctures, by the different lattice mismatch, enables it to apply in spintronics devices.

## Discussions

In summary, the structural, electronic and magnetic properties of the hydroxides (Ni(OH)_2_) and nitrides XN (X = B, Al, and Ga) heterostructures are carefully investigated by first-princiles calculations. The results show that the ground state of the pristine monolayer Ni(OH)_2_ is a semiconductor with antiferromagnetic (AFM) coupling between two nearest Ni atoms. Interestingly, the magnetic coupling of Ni(OH)_2_ can be easily tuned by forming the heterostructure with monolayer XN. It is found that Ni(OH)_2_-GaN and Ni(OH)_2_-AlN heterostructures retain the AFM coupling between two nearest Ni atoms, while Ni(OH)_2_-BN heterostructure have a larger magnetic moment with ferromagnetic (FM) coupling. More interestingly, complete electron–hole separation is found in the Ni(OH)_2_-GaN heterostructure. The versatile electronic properties and tunable magnetic properties of the nickel Ni(OH)_2_-XN heterostructures make it promising candidate for applications in spin-devices.

## Method

The first-principles structure and energy calculations are performed using the Vienna Ab Initio Simulation Package (VASP)^38^,^39^. Projector augmented-wave (PAW) pseudopotentials^40^ were used to account electron-ion interactions. The generalized gradient approximation (GGA) with the PBE functional^41^ was used to treat the exchange-correlation interaction between electrons. A vacuum region larger than 15 Å perpendicular to the sheets (along the **c** axis) is applied to avoid the interaction between layers caused by the periodic boundary condition. In our calculation, a kinetic-energy cutoff for plane-wave expansion is set to 500 eV. All the atoms in the unit cell are fully relaxed until the force on each atom is less than 0.02 eV/Å. Electronic energy minimization was performed with a tolerance of 10^−4^ eV. The Brillouin-zone (BZ) sampling is carried out with a 11 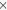 11 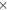 1 Monkhorst-Pack grid for the 2D stacked heterostructures. The vdW interaction is corrected by the DFT-D3 approach^42^.

## Additional Information

**How to cite this article**: Wei, X.-L. *et al*. Electronic and magnetism properties of two-dimensional stacked nickel hydroxides and nitrides. *Sci. Rep*. **5**, 11656; doi: 10.1038/srep11656 (2015).

## Figures and Tables

**Table 1:** The relative energy of different magnetic coupling, ground state geometries, and magnetic moment of Ni(OH)_2_–XN heterostructures.

structures	FM (eV)	AFM1 (eV)	AFM2 (eV)	a (Å)	M_tot_ (μ_B_)
Ni(OH)_2_/BN	0.000	0.061	0.059	2.705	7.914
Ni(OH)_2_/AlN	0.031	0.000	0.003	3.123	0.000
Ni(OH)_2_/GaN	0.056	0.001	0.000	3.202	0.000

Three different magnetic couplings: FM (Ni1↑, Ni2↑, Ni3↑, Ni4↑), AFM1 (Ni1↑, Ni2↓, Ni3↑, Ni4↓), and AFM2 (Ni1↑, Ni2↓, Ni3↓, Ni4↑) are considered in the Ni(OH)_2_–XN heterostructures. Where Ni1-Ni4 atoms are labeled in Fig. 1(b), and the symbol of ↑ and ↓ represent the spin-up and spin-down states, respectively.
